# Proton-Sensing Ovarian Cancer G Protein-Coupled Receptor 1 on Dendritic Cells Is Required for Airway Responses in a Murine Asthma Model

**DOI:** 10.1371/journal.pone.0079985

**Published:** 2013-11-11

**Authors:** Haruka Aoki, Chihiro Mogi, Takeshi Hisada, Takashi Nakakura, Yosuke Kamide, Isao Ichimonji, Hideaki Tomura, Masayuki Tobo, Koichi Sato, Hiroaki Tsurumaki, Kunio Dobashi, Tetsuya Mori, Akihiro Harada, Masanobu Yamada, Masatomo Mori, Tamotsu Ishizuka, Fumikazu Okajima

**Affiliations:** 1 Laboratory of Signal Transduction, Institute for Molecular and Cellular Regulation, Gunma University, Maebashi, Japan; 2 Department of Medicine and Molecular Science, Gunma University Graduate School of Medicine, Maebashi, Japan; 3 Gunma University School of Health Sciences, Maebashi, Japan; 4 Laboratory of Allergy and Immunology, Faculty of Pharmacy, Takasaki University of Health and Welfare, Takasaki, Japan; 5 Department of Cell Biology, Graduate School of Medicine, Osaka University, Osaka, Japan; 6 Third Department of Internal Medicine, Faculty of Medical Sciences, University of Fukui, Fukui, Japan; French National Centre for Scientific Research, France

## Abstract

Ovarian cancer G protein-coupled receptor 1 (OGR1) stimulation by extracellular protons causes the activation of G proteins and subsequent cellular functions. However, the physiological and pathophysiological roles of OGR1 in airway responses remain largely unknown. In the present study, we show that OGR1-deficient mice are resistant to the cardinal features of asthma, including airway eosinophilia, airway hyperresponsiveness (AHR), and goblet cell metaplasia, in association with a remarkable inhibition of Th2 cytokine and IgE production, in an ovalbumin (OVA)-induced asthma model. Intratracheal transfer to wild-type mice of OVA-primed bone marrow-derived dendritic cells (DCs) from OGR1-deficient mice developed lower AHR and eosinophilia after OVA inhalation compared with the transfer of those from wild-type mice. Migration of OVA-pulsed DCs to peribronchial lymph nodes was also inhibited by OGR1 deficiency in the adoption experiments. The presence of functional OGR1 in DCs was confirmed by the expression of OGR1 mRNA and the OGR1-sensitive Ca^2+^ response. OVA-induced expression of CCR7, a mature DC chemokine receptor, and migration response to CCR7 ligands in an in vitro Transwell assay were attenuated by OGR1 deficiency. We conclude that OGR1 on DCs is critical for migration to draining lymph nodes, which, in turn, stimulates Th2 phenotype change and subsequent induction of airway inflammation and AHR.

## Introduction

Allergic asthma is a Th2 lymphocyte-mediated inflammatory airway disease characterized by eosinophilia, increased mucus production by goblet cells, and airway hyperresponsiveness (AHR) [1,2]. These asthmatic responses are mediated by Th2 cytokines, including IL-4, IL-5, and IL-13; IL-5 plays important roles in differentiation, recruitment, and activation of eosinophils and IL-4 and IL-13 are considered to be involved in the driving of IgE synthesis from B-cells, goblet cell metaplasia, and AHR. Macrophages and neutrophils also accumulate in the inflammatory lesion. Dendritic cells (DCs) are antigen-presenting cells and play a central role in adaptive and innate immune responses. Upon antigen exposure at the epithelium, DCs migrate to draining lymph nodes and have been shown to be necessary for the induction of Th2 responses to many antigens by stimulating naïve T cells during sensitization as well as maintaining adaptive Th2 cell response [1,2]. 

It is well known that asthma is associated with airway acidification, reaching pH 5.2 under severe asthmatic conditions [3-5], due to the accumulation of inflammatory cells in peribronchial and perivascular spaces, where stimulation of glycolysis and respiratory burst may cause the production of lactate and protons [6]. Under such a severe acidic pH, the proton-sensing capsaicin-sensitive TRPV1 channel and/or acid-sensing ion channels in sensory nerves have been suggested to be involved in the initiation and development of asthmatic symptoms [4,7]. 

Recent studies have shown that OGR1-family G protein-coupled receptors (GPCRs), including OGR1, G protein-coupled receptor4 (GPR4), and T-cell death associated gene 8 (TDAG8), which were previously proposed as receptors for lysolipids, such as sphingosylphosphorylcholine (SPC), sense extracellular acidification through histidine residue [8-10], resulting in the stimulation of a variety of intracellular signaling pathways through heterotrimeric G proteins [11-13]. OGR1 is coupled with the phospholipase C and Ca^2+^ signaling pathways and mediates a variety of acidic pH-induced actions in airway smooth muscle cells [14-16] and other cell types [17,18]. Moreover, OGR1 has been shown to be expressed in epithelial cells [19], macrophages [20], and neutrophils [21]. Thus, OGR1 is potentially involved in the pathogenesis of asthmatic responses. However, the roles of OGR1 in the induction of cardinal responses in airway inflammation have not yet been reported in vivo. In the present study, we show that OGR1-deficient mice are resistant to the airway eosinophilic inflammation and AHR relative to wild-type (WT) mice at least partly through the change in DC migration activity. 

## Materials and Methods

### Ethics Statement

This study was carried out in strict accordance with the guidelines of the Animal Care and Experimentation Committee of Gunma University, and all animals were bred in the Institute of Animal Experience Research of Gunma University. The protocol was approved by the Animal Care and Experimentation Committee of Gunma University (Permit Number: 11-019). All surgery was performed under sodium pentobarbital anesthesia, and all efforts were made to minimize suffering.

### Mice

We have recently generated OGR1-deficient C57BL/6 mice [22]. Female mice with a targeted disruption of the OGR1 (*OGR1^-/-^* mice) were backcrossed for ten generations onto a BALB/c genetic background. BALB/c *OGR1^-/-^* mice and their littermates (*OGR1^+/+^* or WT) were used at 5–6 wk of age. Mice were housed under specific pathogen-free conditions and maintained on an OVA-free diet. 

### Sensitization and Airway Challenge

*OGR1^-/-^* and *OGR1^+/+^* (or WT) mice were assigned to the following two groups: PBS/PBS group or control group, intraperitoneal sensitization with PBS and airway challenge with PBS, and OVA sensitization/challenge group, intraperitoneal sensitization with OVA and airway challenge with OVA. Sensitization and challenge with OVA were performed as described [23]. In brief, mice were sensitized by injections of 10 μg OVA (Grade V) (Sigma-Aldrich, St. Louis, MO), along with 1 mg of aluminum hydroxide (Alu-Gel-S; SERVA, Heidelberg, Germany) used as an adjuvant in 0.2 ml, on days 0 and 14. Mice were subsequently challenged via the airways by inhalation exposure to aerosols of OVA (1% in PBS) for 20 min on Days 28, 29, and 30. On Day 32, airway responses were evaluated.

### AHR

AHR was measured by both indirect noninvasive and direct invasive methods in mice 48 h after the final OVA challenge. In indirect noninvasive method [24], reactivity to methacholine was assessed in the whole body plethysmograph (Buxco Electronics, Inc, Troy, NY). In brief, saline or methacholine was given by aerosol through an inlet of the chamber for 3 min. Readings were initiated at 3 min and continued for 10 min. Peak values of 5-min average enhanced pause (Penh) were determined. Penh is defined as an evaluation of changes in the shape of the airflow pattern entering and leaving a whole-body flow plethysmograph as an animal breathes. Data are expressed as percent change from baseline values obtained after inhalation of saline. There were no significant differences in baseline values among the different groups. For invasive measurement of lung resistance, mice were anesthetized, tracheostomized, and mechanically ventilated. AHR was assessed by measuring changes in lung resistance (RL) in response to inhaled methacholine, as described previously [25]. Data are expressed as percent change from baseline RL values obtained after inhalation of saline. There were no significant differences in baseline values among the different groups in both methods. 

### Bronchoalveolar Lavage (BAL)

Mice were given a lethal dose of pentobarbital (60 mg/kg i.p.) and the lungs were lavaged with 1 ml aliquots of Hanks’ balanced salt solution (HBSS) through the tracheotomy. The lavage fluid was centrifuged (300 × g for 10 min at 4°C), and the BAL fluid supernatant was stored at -80°C prior to subsequent assay. The cell pellet was resuspended in 1 ml of HBSS. Total cell counts were performed by adding 50 μl of the cell suspension to 50 μl of Kimura stain, and cells were counted in a Neubauer chamber under light microscopy. Differential cell counts were made on cytospin preparations, prepared by centrifuging at 500 rpm for 5 min and staining with May-Grünwald-Giemsa stain. Cells were identified as macrophages, neutrophils, eosinophils, and lymphocytes according to standard morphology. Three hundred cells were counted under × 400 magnification, and the percentage and absolute number of each cell type were calculated.

### Histological Studies

Lung sections from different experimental groups of mice were prepared and analyzed as described previously [26]. Briefly, left lungs were fixed in 4% paraformaldehyde and embedded in paraffin. The sections (4 µm) were stained with periodic acid-Schiff (PAS) for identification of the mucus-secreting cells (goblet cells) of the airways and counterstained with hematoxylin. The stained goblet cells were enumerated in large-caliber preterminal bronchi at least three lung sections obtained from each animal. The length of basal lamina of corresponding bronchus was measured by Image J (National Institutes of Health). The data were expressed as the mean of PAS positive goblet cells in bronchus per millimeter of basement membrane. The degree of peribronchial and perivascular inflammation (Inflammation score) was evaluated on a subjective scale of 0 to 3, as described [27]. Three independent blinded investigators graded the inflammation score. A value of 0 was assigned when no inflammation was detectable, a value of 1 was assigned for occasional cuffing with inflammatory cells, a value of 2 was assigned for most bronchi or vessels surrounded by a thin layer (one to five cells thick) of inflammatory cells, and a value of 3 was given when most bronchi or vessels were surrounded by a thick layer (more than five cells thick) of inflammatory cells. 

### Generation of Bone Marrow-Derived DCs (BMDCs)

DCs were generated from bone marrow cells of WT or *OGR1* ^*-/-*^ mice according to the procedure previously described [28]. In brief, bone marrow cells obtained from femurs and tibias of mice were placed in RPMI 1640 medium (pH 7.4) containing 10% FBS with recombinant mouse GM-CSF (10 ng/ml; R&D Systems) and recombinant mouse IL-4 (10 ng/ml; R&D Systems). The culture medium was changed with the same medium with these cytokines every two days. On day 7, cells were pulsed with 100 µg/ml OVA (Grade V from Sigma-Aldrich) for 24 h in the same culture medium with FBS, GM-CSF, and IL-4. It is noted that the pH 7.4 medium was slightly acidified to 7.2 at 6 h and 7.0 at 24 h after the culture of the cells with OVA in a humidified air/CO_2_ (19:1). The change in medium pH may be in part related to DC maturation by OVA used in the present study, which contains a low amount of LPS [29]. In order to keep the pH steady state, we cultured the cells in the medium containing 20 mM HEPES; however, we noticed that the effect of OVA pulse on the expression of CCR7 is attenuated. We therefore used commercially available RPMI 1640 medium without additional buffer agents in in vitro experiments except for the short term Ca^2+^ response as shown below. When the medium pH was changed, HCl or NaOH was added to the culture medium.

### Adoption Experiment with BMDCs

This was performed as described [30]. In brief, BMDCs prepared from WT or OGR1-deficient mice were sensitized with OVA or its vehicle (PBS) 24 h before adoption experiments. OVA-pulsed BMDCs (1 x 10^6^ cells in 40 µl of PBS) were instilled into WT mice through the trachea. Ten days later, mice were exposed to aerosolized OVA (1% in PBS) for 20 min/day for three consecutive days; 48 h after the last challenge, AHR was assessed and BAL fluid was obtained. Thus, the adoption experiment allowed to detect significant asthmatic responses at 14 days, which is much shorter than the time (32 days) required for the detection of airway responses by the regular in vivo OVA sensitization/challenge protocol. This may be due to the in vitro effective sensitization of DCs with OVA in the DC adoption experiments.

### BMDC Migration Assay in vivo

To study the distribution of injected BMDCs, cells were prepared from WT or OGR1-deficient mice and pulsed with PBS or OVA as described above. The cells were labeled with 10 μg/ml carboxyfluorescein diacetate succinimidyl ester (CFSE) (Molecular probes, Inc., Eugene, OR) for 10 min at 37°C in RPMI 1640 containing 0.5% BSA (Fraction V) (Sigma-Aldrich). Four groups of labeled cells (PBS-treated WT DC, PBS-treated OGR1-deficient DC, OVA-treated WT DC, and OVA-treated OGR-1 deficient DC) were instilled intratracheally into 3 naïve WT mice per each group. The peribronchial lymph nodes from 3 mice were collected per each group and CFSE^+^ DCs migrating in the peribronchial lymph nodes during 96 h were analyzed by flow cytometry, as described previously [30]. Similar experiments were performed 3 times. Migration activity was expressed as percentage of CFSE^+^DCs (CFSE^+^CD11c^+^) per total cells applied. 

DC migration activity was further evaluated by a footpad assay, in which OVA-pulsed BMDCs were labeled with CFSE as described and cells were injected subcutaneously into the footpad of WT mice. The migration of the cells into popliteal lymph nodes were analyzed by flow cytometry 24 h after the injection of the cells. 

### Measurement of Cytokines and IgE

Cytokine levels in the BAL fluid were measured by Bio-Plex Suspension Array System (Nippon Bio-Rad Laboratories, Tokyo, Japan) for IFN-γ, IL-4, and IL-5 and by ELISA kit (eBioscience, San Diego, CA) for IL-13, according to the manufacturer's instructions. Plasma levels of OVA-specific IgE were measured by DS mouse IgE ELISA (OVA) kit (DS Pharma Biomedical, Osaka, Japan), as described previously [23].

### Measurement of mRNAs

Total RNA was isolated and reverse-transcribed using random priming and Multiscribe reverse transcriptase (Applied Biosystems, Foster City, CA). The mRNAs were measured by a quantitative real-time TaqMan PCR with Mx3000P Real-time PCR System (Agilent Technologies, Santa Clara, CA) using specific probes for OGR1 (Mm01335272), TDAG8 (Mm00433695), GPR4 (Mm00558777), G2A (Mm00490809), CCR7 (Mm01301785), and GAPDH (4352932E), as described previously [14].

### Measurement of Intracellular Ca^2+^ Concentration

The non-pulsed BMDCs were harvested from 10-cm dishes with 4 mM EDTA/PBS and labeled with fura2/AM. Intracellular Ca^2+^ concentration ([Ca^2+^]_i_) was measured in cell suspension under gently stirring condition in HEPES-buffered medium based on the change in fura-2 fluorescence as described previously [14]. The HEPES-buffered medium consisted of 20 mM HEPES (pH 7.4), 134 mM NaCl, 4.7 mM KCl, 1.2 mM KH_2_PO_4_, 1.2 mM MgSO_4_, 2 mM CaCl_2_, 2.5 mM NaHCO_3_, 5 mM glucose, and 0.1% BSA. The pH of the medium was adjusted by addition of an appropriate amount of HCl.

### Flow Cytometry

BMDCs were stained with PE-conjugated monoclonal antibodies (mAbs) diluted in PBS containing 1% BSA (FACS buffer). The PE-conjugated Abs, including anti-CD11c and anti-CCR7, and isotype control were obtained from BD Biosciences, San Diego, CA. After incubating with Abs for 10 min at 4°C, cells were washed twice with FACS buffer and analyzed by ®FACScan flow cytometer using CellQuest software (Becton Dickinson, Mountain View, CA).

### In vitro Migration Assay

On day 7, WT or *OGR1* ^*-/-*^ BMDCs were incubated with or without OVA (100 μg/ml) as described above. After 24 h, cultured cells were harvested and resuspended in RPMI 1640 medium at pH 7.4 with 0.1% BSA. Cells (4 x 10^5^) were added on top of a Transwell culture insert with a 8-mm diameter and 5-µm pore size chemotaxicell (Kurabo, Osaka, Japan). The test agents were added to the lower compartment of 24-well plates. The number of cells migrated to the lower chamber within 2.5 h was determined by fluorescence change with CyQUANT GR Dye (Molecular probes, Inc., Eugene, OR) after lysis of the cells.

### Statistical Analysis

All experiments were performed in duplicate or triplicate. The results of multiple observations are presented as the mean + SEM or as representative results from more than three different batches of cells, unless otherwise stated. In vivo experiments, we usually performed four to six mice per group in each experiment and carried out two or three times the same experiments. For the presentation of the results of in vivo study, we usually combined all the results, unless otherwise stated. All statistical calculations were performed with GraphPad Prism 5 (La Jolla, Calif). ANOVA was used to determine the levels of difference between all groups. Comparisons for all pairs were performed by unpaired Student *t* test. A value of *p* < 0.05 was considered significant. 

## Results

### Generation of OGR1^-/-^ BALB/c Mice

**Figure 1:**
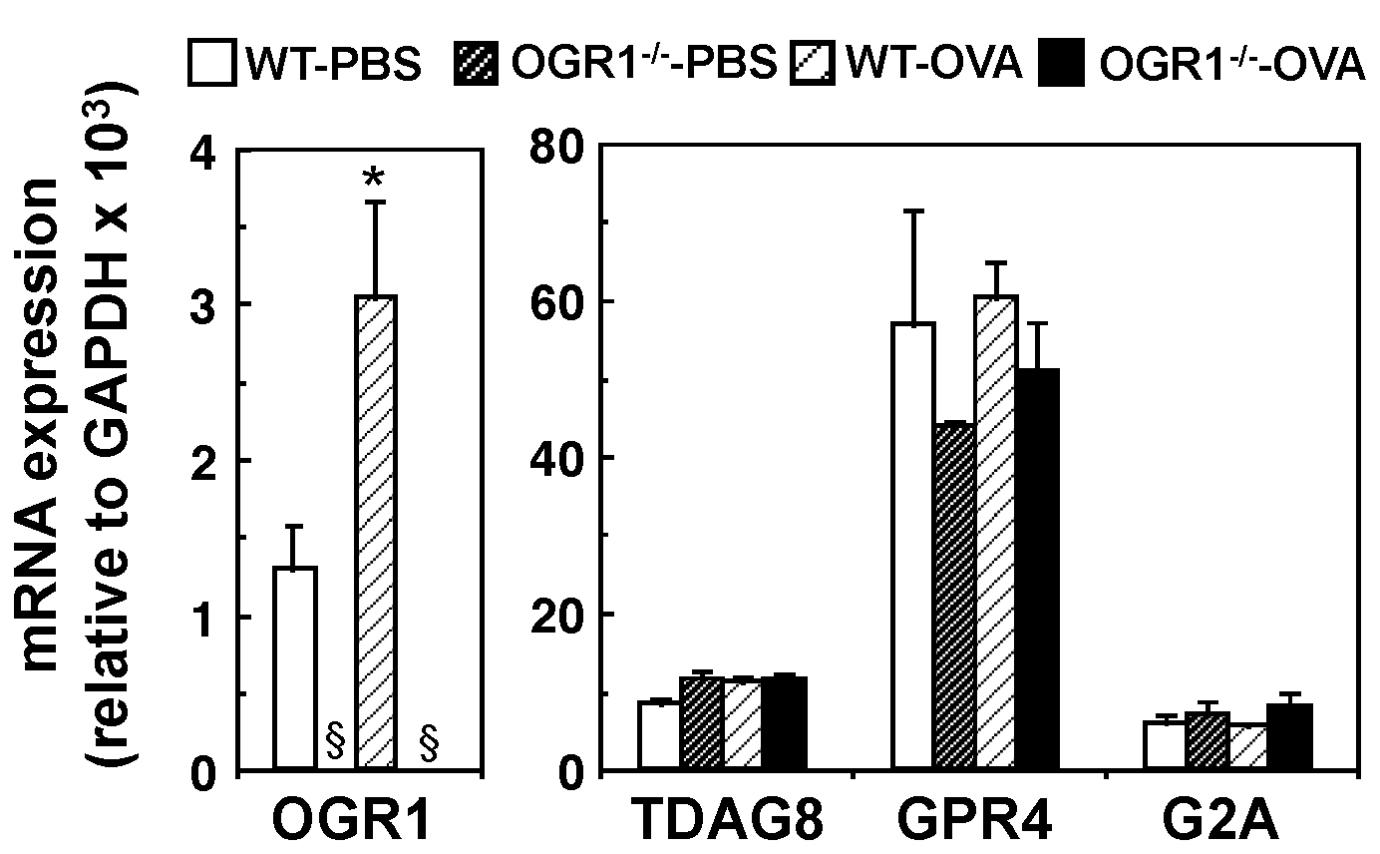
OGR1 mRNA expression in lung is increased by OVA sensitization/challenge. WT and *OGR1^-/-^* mice were sensitized by OVA/alum and challenged by OVA. To nonsensitized mice, PBS was injected and inhaled. Lung mRNA expression of OGR1 and other proton-sensing GPCRs was measured 48 h after last antigen challenge. The results are mean + SEM of 9 determinations from three separate experiments. *The effect of OVA-priming (WT-PBS vs. WT-OVA) was significant. ^§^The expression of OGR1 mRNA was undetectable.

**Figure 2:**
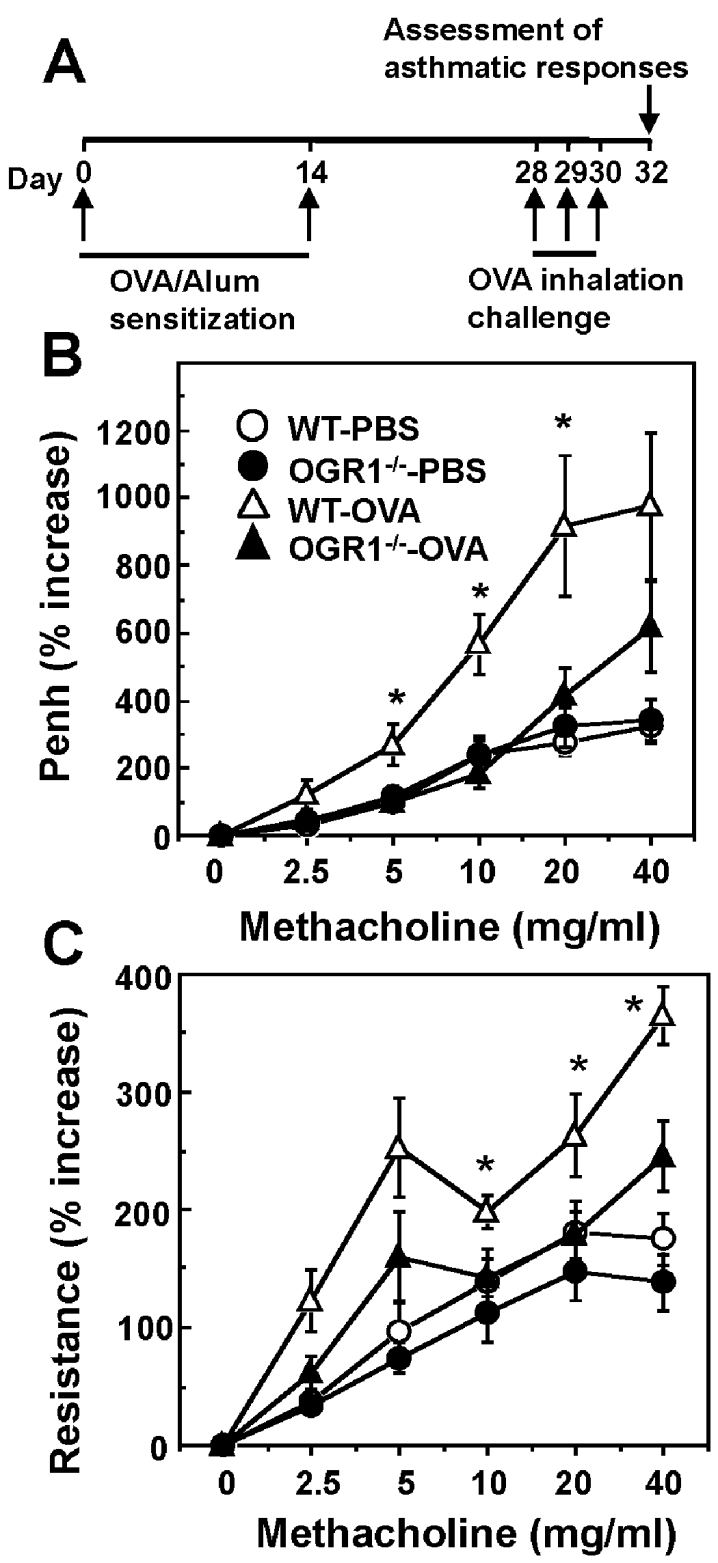
OGR1 deficiency attenuates AHR to methacholine in OVA-sensitized mice. (A) OVA sensitization and challenge protocol. WT and *OGR1* ^*-/-*^ mice were sensitized by i.p. injection of OVA/alum and challenged by OVA aerosol (triangle). To nonsensitized mice, PBS was injected and inhaled (circle). AHR to the inhaled methacholine was assessed by changes in noninvasive Penh (B) and dynamic resistance (C) 48 h after the last antigen exposure. Data are mean + SEM of *n*=13-16 per group. *p < 0.05 (WT-OVA vs. OGR1^-/-^-OVA).

To understand the role of OGR1 in asthmatic airway inflammation, we created OGR1-deficient BALB/c mice. OGR1 mRNA expression is observed in lungs from WT mice, although the expression levels are not as high as those of other proton-sensing GPCRs (Figure 1). In the bronchial asthma model, mice were sensitized by intraperitoneal injection of OVA in alum adjuvant twice and challenged with OVA aerosol three times (see Figure 2A), resulting in 2.5 times increase in OGR1 mRNA expression in lungs (Figure 1). Such a significant increase in mRNA expression was not observed in other proton-sensing GPCRs. As expected, the OGR1 mRNA expression was completely lost, whereas other OGR1 family GPCR mRNA expression was unchanged, in the lungs of *OGR1^-/-^* mice regardless of the OVA treatment (Figure 1).

### OGR1 Deficiency Attenuates AHR and Airway Inflammation in OVA-Sensitized Mice

The pathophysiological roles of OGR1 in bronchial asthma were characterized using the OVA asthma model (Figure 2A). We first assessed an increase in enhanced pause (Penh) by using barometric whole-body plethysmography as an index of airway contraction in response to increasing doses of methacholine (Figure 2B). Inhaled aerosolized methacholine is known to induce bronchoconstriction. OVA sensitization/challenge clearly increased Penh in response to methacholine in WT mice; however, the AHR to OVA sensitization/challenge was significantly attenuated in OGR1-deficient mice to the level of that of mice sensitized and challenged with PBS alone (Figure 2B). Since the measurement of Penh as AHR is controversial [31-33], we measured lung resistance with a direct invasive method as well. Consistently with the results of Penh, OVA sensitization/challenge increased the lung resistance in response to methacholine in WT mice and OGR1 deficiency attenuated the OVA-induced increase in lung resistance to the level of that of control mice sensitized and challenged with PBS (Figure 2C). 

**Figure 3:**
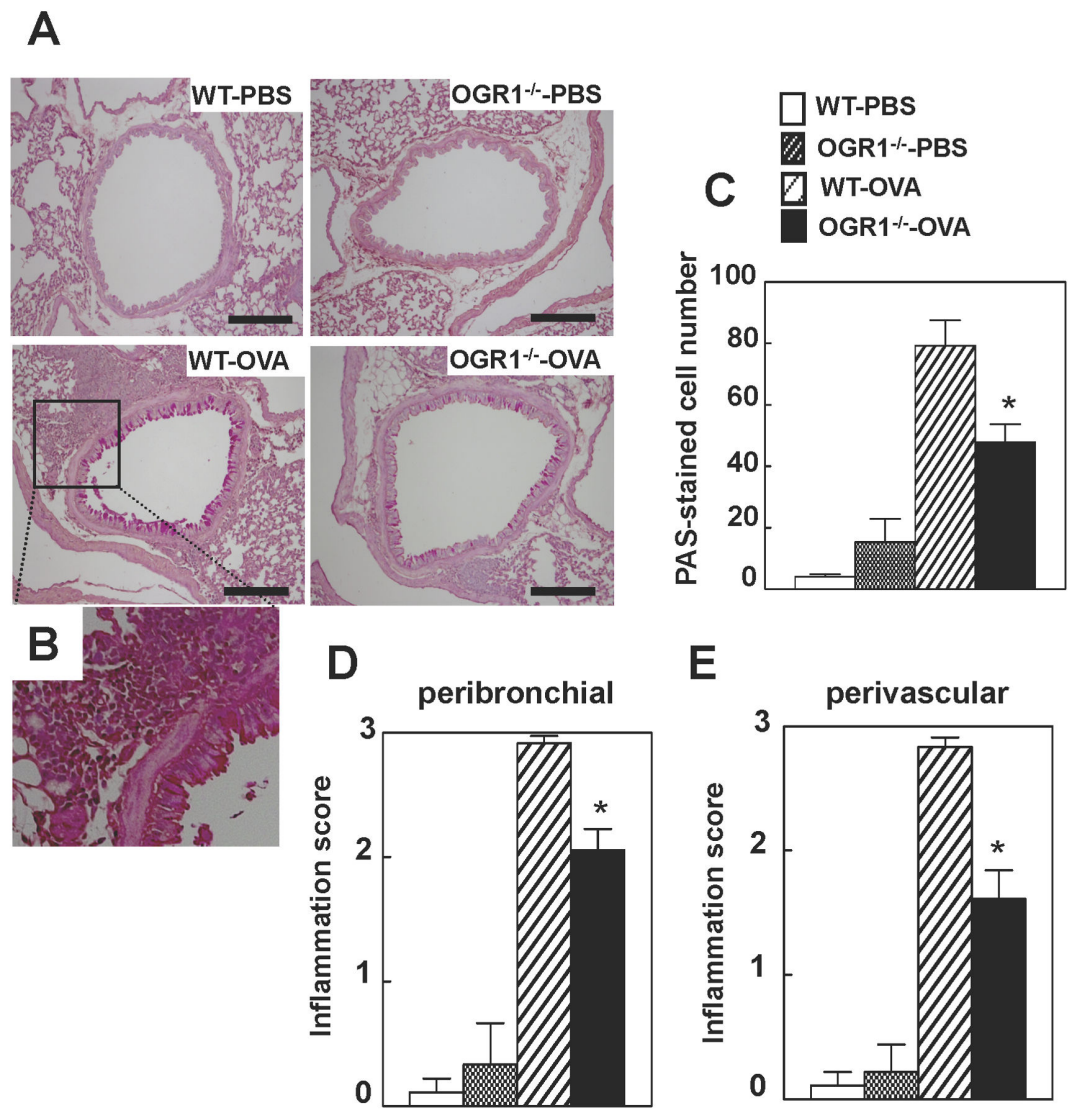
Histological analysis of lung sections. Mice were sensitized and challenged by OVA or PBS as described in Figure 2. (A) Histological analysis of lung sections was performed 48 h after the last antigen exposure. The hematoxylin and PAS-stained sections shown are representative of three lung sections per mouse from 5 to 6 mice in each group. Scale bar, 200 μm. (B) The higher magnification image of the square in WT-OVA is shown. (C) Goblet cell hyperplasia was quantified as a PAS-staining cell number (C), and degree of peribronchial inflammation (D) and perivascular inflammation (E) was evaluated on a subjective scale of 0 to 3 as inflammatory score, in using the same sections as those used in (A). The column design specifying each group in (D) and (E) is the same as those in (C). Effects of OGR1 deficiency (WT-OVA vs. OGR1^-/-^-OVA) were significant (*p < 0.05) throughout the figure.

OVA sensitization/challenge caused dense peribronchial and perivascular accumulation of inflammatory cells as well as metaplasia and mucus hyperproduction of airway walls in WT mice (Figure 3A and B). These changes were minimal in OGR1-deficient mice with OVA sensitization/challenge (Figure 3A). The degree of goblet cell metaplasia and mucus hyperproduction was assessed by PAS staining, which was quantified as PAS-stained cell numbers per length of basal lamina (mm) (Figure 3C). Stimulated PAS staining by OVA sensitization/challenge was significantly inhibited by OGR1 deficiency. As for the peribronchial and perivascular inflammation, the degree of the accumulation of inflammatory cells was assessed as inflammation score (Figure 3D and E), which showed that OGR1-deficiency significantly inhibited OVA sensitization/challenge-induced inflammatory cell accumulation. These results suggest that OGR1 is involved in goblet cell hyperplasia and peribronchial and perivascular inflammation.

**Figure 4:**
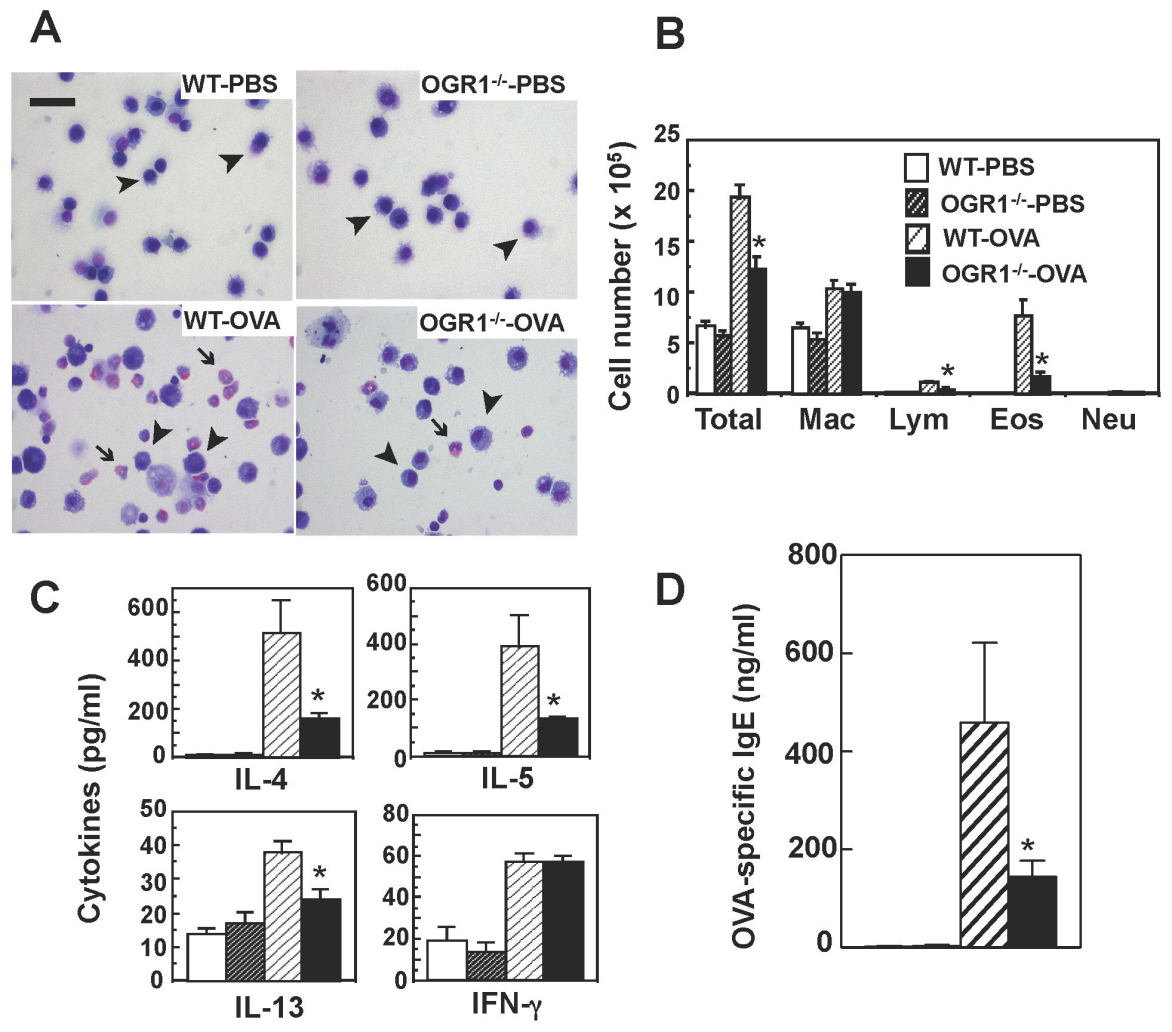
OGR1 deficiency attenuates infiltration of inflammatory cells, Th2 cytokine levels in BAL fluids, and plasma IgE levels in OVA-sensitized mice. Mice were sensitized and challenged by OVA or PBS as described in Figure 2. BAL fluid was analyzed 48 h after the last antigen exposure for total cell count and profile of cell types. Representative images of inflammatory cells (A) and differential cell counts (B) in BAL fluids. Arrowheads and arrows represent macrophages and eosinophils, respectively. Scale bar, 200 μm. Mac, macrophage; Lym, lymphocyte; Eos, eosinophils; and Neu, neutrophils. Data are mean + SEM of *n*=10-12 per group. The same BAL fluids were used to measure IL-4, IL-5, IL-13, and IFN-γ (C). Data are mean + SEM of *n*=10-12 per group. Plasma was collected for measurement of OVA-specific IgE levels after BAL fluid collection (D). Data are mean + SEM of *n*=6-15 per group. The column design specifying each group in (C) and (D) is the same as those in (B). The effects of OGR1 deficiency (WT-OVA vs. OGR1^-/-^-OVA) were significant (*p < 0.05) throughout the figure.

We analyzed the numbers and population of the cells present in BAL fluids (Figure 4A and B). The increased total cell numbers in mice with OVA sensitization/challenge were significantly reduced in OGR1-deficient mice relative to WT mice. Differential cell counts revealed the prominent recruitment of eosinophils into BAL fluids in WT mice with OVA sensitization/challenge, which was remarkably reduced in OGR1-deficient mice with the same treatments. The content of lymphocytes in BAL fluids was also significantly reduced by OGR1 deficiency. There was no significant effect of OGR1 deficiency on the number of macrophages and neutrophils. 

To assess the mechanisms of reduced AHR, eosinophilia, and goblet cell metaplasia, Th1/Th2 cytokines in BAL fluids were measured by ELISA (Figure 4C). OVA sensitization/challenge induced significant increases in Th2 cytokines, including IL-4, IL-5, and IL-13, and Th1 cytokine IFN-γ in WT mice. The Th2 cytokine content in BAL fluids was significantly lower in OGR1-deficient mice than in WT mice. On the other hand, the OVA sensitization/challenge-induced increase in IFN-γ production was not affected by OGR1 deficiency (Figure 4C). Plasma levels of IgE specific to OVA were also inhibited by OGR1 deficiency in OVA sensitization/challenged mice (Figure 4D).

### DCs Express Functional OGR1

**Figure 5:**
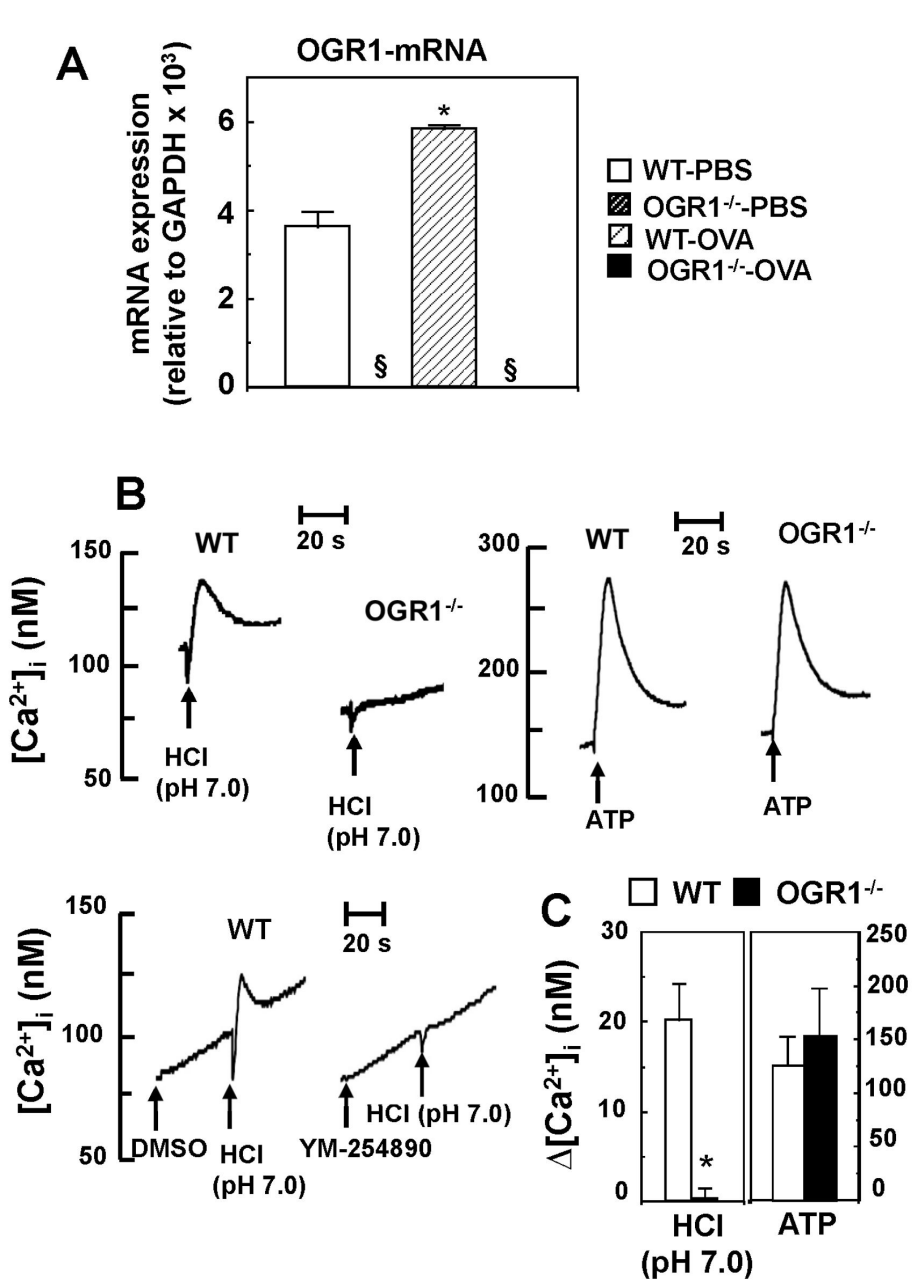
DCs express OGR1 mRNA and functional OGR1 activity. DCs express OGR1 mRNA and functional OGR1 activity. Expression of OGR1 mRNA in DCs and its increase by OVA-priming (A). The expression of OGR1 mRNA was evaluated by quantitative real-time TaqMan PCR. Data are mean ± SEM of 9 determinations from three separate experiments. Effect of OVA-priming (WT-PBS vs. WT-OVA) was significant (*p < 0.05). ^§^The expression of OGR1 mRNA was undetectable. Acidification induces [Ca^2+^]_i_ increase through the OGR1/G_q/11_-protein in DCs (B and C). DCs were prepared from WT and *OGR1* ^*-/-*^ mice and incubated to monitor [Ca^2+^]_i_ evaluated by the change in Fura-2 fluorescence. The cells were preincubated in suspension at pH 7.4 and, at arrow, HCl (final pH of 7.0) or ATP (100 μM) was added. To evaluate the role of G_q/11_-protein, the cell suspension was treated with YM-254890 (100 nM) (or DMSO as vehicle). A representative trace of [Ca^2+^]_i_ change was shown (B). Net [Ca^2+^]_i_ change (peak value - basal value) was evaluated (C). Basal value of [Ca^2+^]_i_ at pH 7.4 was 125 ± 12 nM and the value was not appreciably affected by OGR1 deficiency. Data are mean ± SEM of 8 determinations from four separate experiments. Effect of OGR1 deficiency (WT vs. OGR1^-/-^) was significant (*p < 0.05).

The in vivo results described above suggest that OGR1 plays a role in the process or upstream process of induction of Th2 response in the pathogenesis of airway asthma. We speculate that DC functions are regulated by OGR1. There has been no previous report concerning OGR1 expression and its functions in DCs. Since antibodies specific to OGR1 were not available, the OGR1 expression level was evaluated by mRNA measurement (Figure 5A). We confirmed OGR1 mRNA expression assessed by real-time TaqMan PCR in immature BMDCs of WT mice. Interestingly, mRNA expression of OGR1 was slightly but significantly increased by OVA sensitization (Figure 5A). As expected, OGR1 mRNA expression was completely lost in DCs regardless of OVA sensitization in *OGR1* ^*-/-*^ mice (Figure 5A). We also measured the expression of mRNAs of other proton-sensing GPCRs in DCs. In contrast to lung samples (Figure 1), GPR4 expression was very low in DCs regardless of the OVA pulse (Figure S1). On the other hand, similarly to OGR1, TDAG8 and G2A mRNA expression was significantly increased by OVA sensitization (Figure S1).

To further confirm the functional OGR1 expression in DCs, Ca^2+^ response to extracellular protons was examined because OGR1 is known to be coupled with the G_q/11_ protein/phospholipase C/Ca^2+^ signaling pathways in various types of cells [8,13-19]. Acidification of the extracellular medium to pH 7.0 clearly increased the intracellular Ca^2+^ concentration ([Ca^2+^]_i_), which was almost completely inhibited by OGR1 deficiency and YM-254890, a specific inhibitor for G_q/11_ proteins (Figure 5B and C). The [Ca^2+^]_i_ response to ATP, a ligand for G_q/11_ protein-coupled P2Y-type purinergic receptors, was not affected by OGR1 deficiency (Figure 5B and C). Thus, *OGR1* ^*-/-*^ mice did not show a general defect in G_q/11_ protein signaling. Considered together, these results suggest that DCs express functional OGR1 coupling to G_q/11_ proteins. 

### Intratracheal Transfer of OGR1-Deficient DCs Develops Lower AHR and Eosinophilia Compared with that of WT DCs

**Figure 6:**
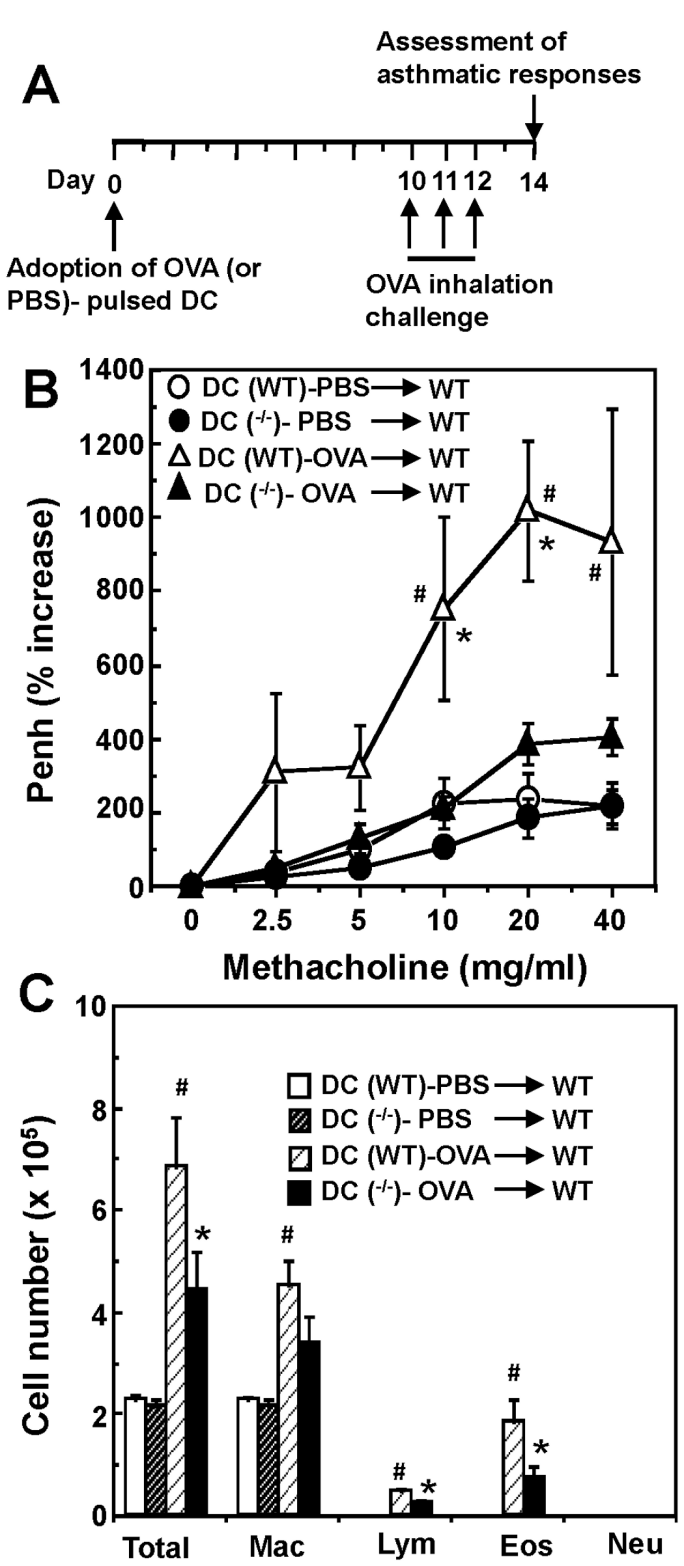
Intratracheal administration of OGR1-deficient DCs showed the lowered AHR and eosinophilia compared with that of WT DCs. (A) Protocol of DC adoption experiment. OVA or PBS-pulsed BMDCs were prepared from WT and *OGR1* ^*-/-*^ mice and administered intratracheally to WT mice. Ten days later, mice were challenged with OVA inhalation for 20 min on three consecutive days. Forty-eight h after the last OVA-challenge, AHR to methacholine was measured as Penh activities (B). After measurement of airway responsiveness, BAL fluids were collected and analyzed for the count of total cells and differentiated cells (C). Data are mean + SEM of *n*=10-12 per group. ^#^ *p* < 0.05 (DC (WT)-PBS vs. DC (WT)-OVA) and *p < 0.05 (DC (WT)-OVA vs. DC (^-/-^)-OVA).

To assess the critical role of OGR1 on DCs in the pathogenesis of asthma, adoptive OVA-pulsed DC transfer experiments were performed. The OVA-pulsed or non-pulsed DCs were intratracheally transferred to WT mice, which was followed by OVA inhalation 10 days after DC administration (Figure 6A). AHR measured as increases in Penh to inhaled methacholine was significantly higher in the intratracheal transfer of OVA-pulsed DCs than that of non-pulsed DCs when DCs were prepared from WT mice (Figure 6B). In contrast, mice receiving OVA-pulsed OGR1-deficient DCs developed lower increases in Penh compared with those that received OVA-pulsed WT DCs.

We also measured the numbers and types of inflammatory cells in BAL fluids of mice transferred with DCs (Figure 6C). The number of eosinophils and lymphocytes in the BAL fluids of mice that received OVA-pulsed OGR1-deficient DCs was significantly lower than those of the mice that received OVA-pulsed WT DCs. These results indicate that the alteration of DC functions may explain, at least in part, the reduced AHR and airway inflammation in *OGR1* ^*-/-*^ mice. 

### OGR1 Deficiency Attenuates Migration of DCs to the Draining Lymph Nodes

**Figure 7:**
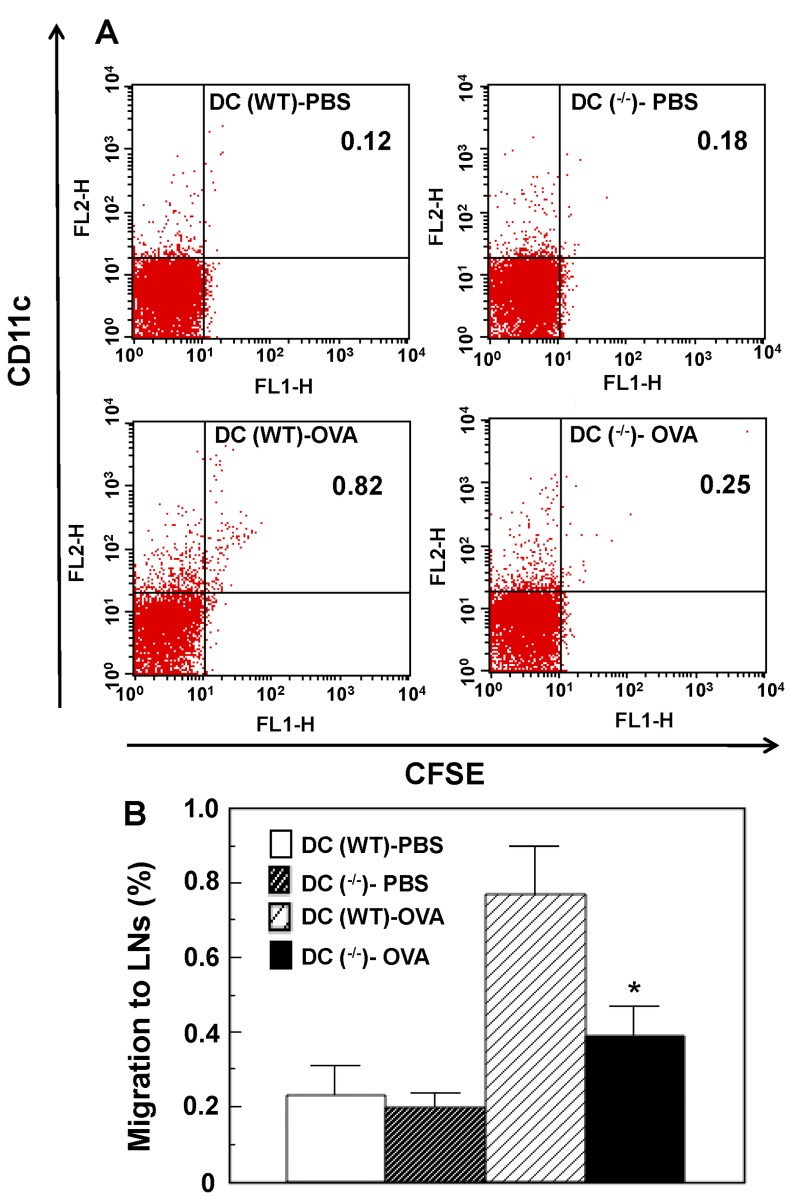
DC migration to peribronchial lymph nodes is inhibited by OGR1 deficiency. Four groups of CFSE-labeled DCs, i.e., PBS-treated WT DC, PBS-treated OGR1-deficient DC, OVA-treated WT DC, and OVA-treated OGR1-deficient DC, were instilled intratracheally to naïve WT mice and the CFSE^+^ DCs migrating in the peribronchial lymph nodes 96 h later were analyzed by flow cytometry. (A) Cells recovered from lymph nodes were assessed for the expression of CD11c and CFSE. Representative flow cytometry plots from 3 separate experiments per group are shown. (B) Percentages of CFSE^+^DCs (CFSE^+^CD11c^+^) per total cells applied were shown. Data are mean ± SEM of 6 analyses from 3 separate experiments. Effect of OGR1 deficiency (DC (WT)-OVA vs. DC (^-/-^)-OVA) was significant (*p < 0.05).

Transferred DCs may have migrated to draining lymph nodes to elicit Th2-mediated AHR and airway infiltration in the adoptive OVA-pulsed DC transfer experiments. The ability of DCs to migrate to peribronchial lymph nodes (PBLNs) was examined. To this end, fluorescently labeled OVA-pulsed DCs derived from WT or *OGR1^-/-^* mice were injected to WT mice intratracheally, and then accumulation of CFSE^+^ DCs migrating in the PBLNs was measured. Under the conditions, migration activity of OVA-pulsed DCs to PBLNs was significantly attenuated by OGR1 deficiency, whereas PBS-pulsed DC migrated to the draining LNs with a low activity regardless of OGR1 expression (Figure 7 A and B).

To prove that the alteration of the migratory activity of DCs by OGR1 deficiency was not restricted to lung draining lymph nodes, CFSE-labeled OVA-pulsed DCs prepared from WT or *OGR1^-/-^* mice were injected subcutaneously into the footpads. Twenty-four hours after the injection, DCs that migrated into the regional lymph nodes or popliteal lymph nodes were analyzed by flow cytometry. OGR1 deficiency significantly suppressed the migratory activity of DCs to popliteal lymph nodes (Figure S2). 

### Attenuation of Migratory Activity of Mature DCs by OGR1 Deficiency in vitro

**Figure 8:**
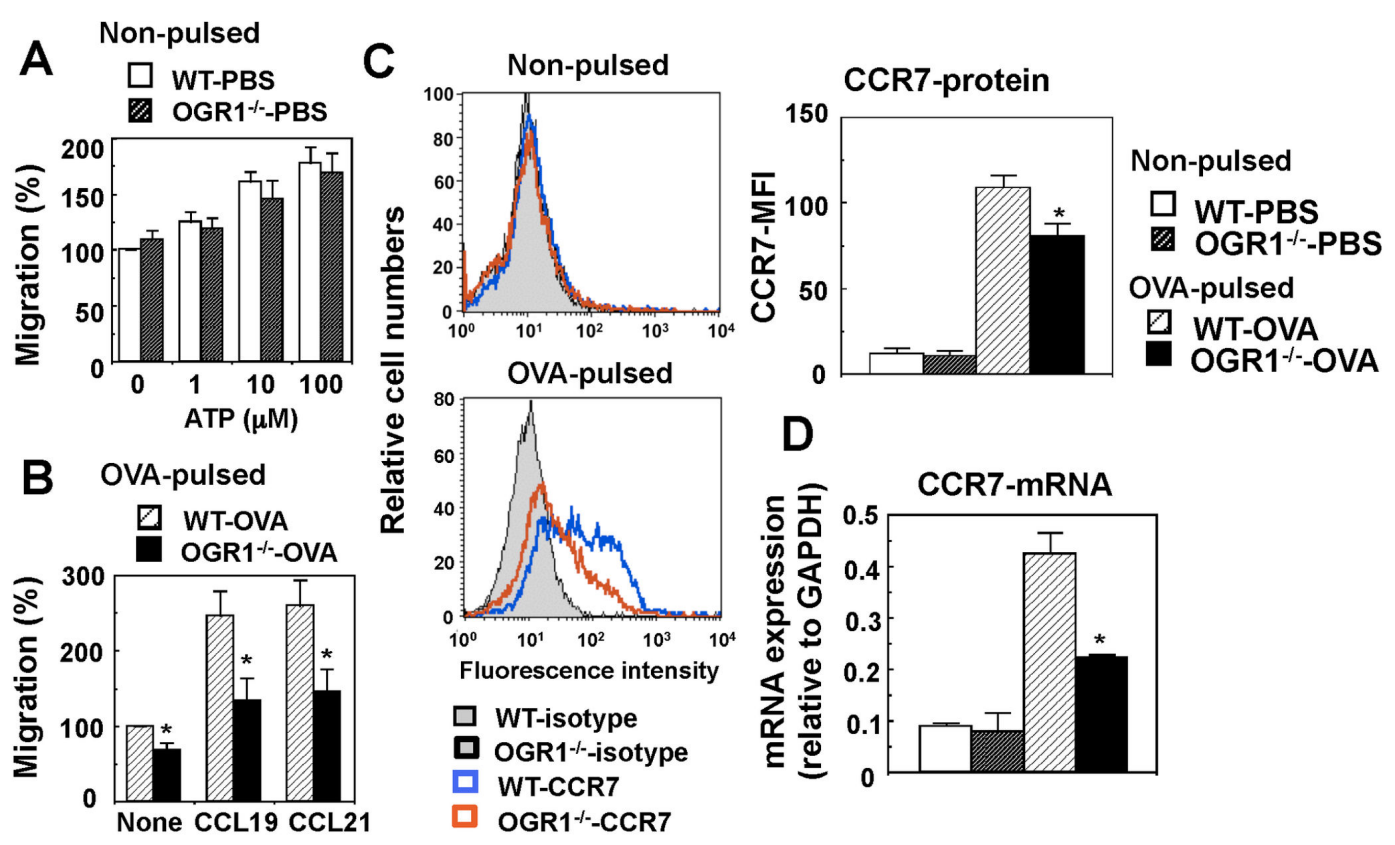
Mature DC migration is attenuated in association with reduced expression of CCR7 by OGR1 deficiency in vitro. (A and B) BMDCs from WT or OGR1-deficient mice were pulsed with 100 μg/ml of OVA as mature DCs or its vehicle as immature DCs overnight. Migration response to the indicated concentrations of ATP for non-pulsed immature DCs (A) and to 200 ng/ml CCL19 or CCL21 for OVA-pulsed DCs (B) was measured. Results are expressed as percentages of basal values obtained without any agonist in DCs of WT mice. Data are mean ± SEM of 5 separate experiments. (C) The expression of CCR7 protein was analysed by flow cytometry. Representative results of four separate experiments in each group are shown. CCR7-protein expression is also expressed as mean fluorescence intensity (MFI) + SEM. (D) The expression of CCR7 mRNA was measured by quantitative real-time TaqMan PCR. Data are mean ± SEM of 9 determinations from three separate experiments. The effects of OGR1 deficiency (WT-OVA vs. OGR1^-/-^-OVA) were significant throughout the figure (*p < 0.05).

The role of OGR1 in the migratory activity of DCs was examined in an in vitro Transwell assay. Although the migratory response to ATP in non-pulsed immature DCs was not affected by OGR1 deficiency (Figure 8A), the migratory activity of OVA-pulsed mature DCs to CCL19 and CCL21 was significantly lower in DCs from *OGR1* ^*-/-*^ mice than in those from WT mice (Figure 8B). Consistently with the reduced migratory activity to CCL19 and CCL21 in mature DCs, the expression level of CCR7, a receptor for these chemokines, was decreased by OGR1 deficiency in protein (Figure 8C) and mRNA (Figure 8D) levels. These results cumulatively suggest that the migration of mature DCs is regulated by OGR1 through the expression of CCR7.

## Discussion

In the present study, we showed for the first time that OGR1 plays a critical role in the induction of asthmatic AHR and inflammation in an OVA-induced experimental asthma model in mice at least in part through the modulation of DC functions. The following observations support this conclusion. First, inhaled OVA-induced asthmatic responses, including eosinophilia, AHR, inflammatory cell accumulation in peribronchial and perivascular space, and goblet cell formation, were all markedly suppressed in OGR1-deficient mice compared with WT mice. The OGR1 deficiency also caused the inhibition of the production of Th2 cytokines and antigen-specific IgE, suggesting that OGR1 is involved in the process or upstream process of the induction of Th2 in the pathogenesis of asthmatic development. Second, intratracheal administration of OVA-primed WT BMDCs to WT mice caused AHR and BAL fluid eosinophilia in response to inhaled OVA. However, the responses to OGR1-deficient DCs were remarkably attenuated compared with those to WT DCs. Third, functional OGR1 is expressed in DCs, as evidenced by OGR1 mRNA expression and the Ca^2+^ response to extracellular acidification. Both activities were completely lost in *OGR1^-/-^* DCs. Finally, the migration activity of mature DCs was impaired by OGR1 deficiency in vivo and in vitro. The inhibition of migratory activity was associated with the reduction of the expression of CCR7, a receptor of chemokines, e.g., CCL19 and CCL21, for mature DCs. Once activated, DCs migrate to draining lymph nodes where they stimulate naïve T cells during sensitization and/or Th2 effector cells to induce their proliferation and Th2 cytokine production. Together, these results strongly suggest that OGR1 in DCs plays a critical role in their migratory activity and thereby induces Th2 phenotype change and subsequent induction of airway inflammation and AHR.

In the DC tracheal transfer experiment, cells were sensitized with OVA in the pH 7.4 medium at the start of incubation; however, the medium pH was decreased to around pH 7.0 during the 24-h culture with OVA probably due to the activation of metabolic activity. Thus, DCs were stimulated with OVA under the conditions of pH 7.4 to 7.0. The OVA-pulsed DCs were then intratracheally administered to mice, which caused severe cardinal asthmatic inflammatory responses in the airway in a manner depending on OGR1. The migration responses of DCs in vitro and in vivo were performed with OVA-pulsed DCs prepared in a similar way. It is noted that the expression level of CCR7 was attenuated rather than stimulated by acidification of initial pH 7.0, which reach about pH 6.5 during the 24-h OVA pulsing; the inhibitory acidic effect was not affected by OGR1 deficiency (data not shown). Thus, drastic extracellular pH change seems to be unnecessary or even inhibitory for the maturation and migration of DCs. Nevertheless, as described above, the migration response of mature DCs in vitro was significantly attenuated by OGR1 deficiency, which was associated with the reduction of CCR7 expression. These results strongly suggest that OGR1 is active at a physiological or mildly acidic pH, resulting in the induction of CCR7 expression and subsequent DC migration. This is not surprising because OGR1-expressing CHO and HEK cells have been shown to be dramatically activated from pH 7.8 to 7.0 [8,34]. Thus, OGR1 on DC plasma membranes may be activated at pH 7.4 to 7.0 (or by the proton concentrations of 40 to 100 nM) and thereby stimulates the expression of CCR7 during OVA priming and subsequent migration to the draining lymph nodes. A similar activation of OGR1 by neutral or mildly acidic pH of 7.4 to 7.0 was observed for glucose-stimulated insulin secretion from islets [22]. The involvement of OGR1 in the migratory activity of DCs is consistent with the results with purinergic P2Y_2_ receptors [35] and leukotriene BLT-1 receptors [30], both of which are known to be coupled with G_q/11_ proteins, like OGR1, and suggested to be involved in the stimulation of the migration of DCs through the expression of CCR7. 

Although our asthma model suggests that the stimulation of OGR1 in DCs by a physiological or mildly acidic pH is critical for the onset of asthmatic responses by regulating their migration, an earlier step in asthmatic responses, this does not mean that OGR1 is not involved in the exacerbation of asthma by severe airway acidification, which has been shown to be associated with the late phase of an asthmatic situation [3-5]. Under such a severe acidic pH condition, in addition to the TRPV1 channel and/or acid-sensing ion channels on sensory nerves [4,7], OGR1 and other proton-sensing GPCRs in airway smooth muscle cells, epithelial cells, eosinophils, and other cell types may be activated and partly contribute to some features of asthmatic airway inflammation. Indeed, extracellular acidification to around pH 6.3 increased the production of IL-6 and connective tissue growth factor (CTGF), both of which are thought to be involved in the exacerbation of asthma and remodeling of airways, and induced Ca^2+^ mobilization and cell contraction through OGR1 in human airway smooth muscle cells in vitro [14-16]. Thus, AHR to methacholine may be explained in part by the acidic pH-induced contraction of airway smooth muscle cells through OGR1. Apart from OGR1, TDAG8, another type of OGR1 family GPCR that couples with the cAMP pathway, is activated by an acidic pH of 7.0 to 6.0, resulting in the inhibition of eosinophil apoptosis and the exacerbation of airway inflammation of eosinophilia [36], inhibition of inflammatory cytokine production in macrophages [20], and inhibition of superoxide anion production in neutrophils [21]. Moreover, T lymphocytes express both OGR1 and TDAG8, although their roles have not yet been characterized. Thus, although further experiments are undoubtedly required to know the precise roles of proton-sensing GPCRs in the pathogenesis of allergic asthma, the present study has clearly shown that OGR1 on DCs is one critical molecule for regulating asthma development. The intracellular signaling mechanism by which OGR1 stimulation leads to the increased expression of CCR7 is currently being investigated in our laboratory. 

In conclusion, the present study demonstrates that OGR1 is involved in Th2-mediated cardinal asthmatic inflammation and AHR at least partly through the alteration of DC functions, especially migration activity. Thus, OGR1 is a potential target for the treatment of allergic asthmatic inflammation. 

## Supporting Information

Figure S1 **Expression profile of proton-sensing GPCR mRNA in DCs.** BMDCs derived from WT or OGR1-deficienct mice were pulsed with PBS or OVA, and expression of TDAG8, GPR4, and G2A mRNAs was evaluated by a quantitative real-time TaqMan PCR. Data are mean ± SEM of three separate experiments. Effect of OVA-priming (WT-PBS vs. WT-OVA) was significant (^#^ *p* < 0.05).
(TIFF)

Figure S2 **DC migration to popliteal lymph nodes is attenuated by OGR1 deficiency in vivo.** CFSE-labeled OVA-pulsed BMDCs from WT and *OGR1* ^*-/-*^ were subcutaneously injected to footpads of WT mice. Twenty-four h later, the CFSE^+^ DCs migrating in draining lymph nodes was analyzed by flow cytometry. (A) Cells recovered from the lymph nodes were assessed for the expression of CD11c and CFSE. Representative flow cytometry plots from 4 separate experiments per group are shown. (B) Percentages of CFSE^+^DCs (CFSE^+^CD11c^+^) per total cells applied were shown. Data are mean ± SEM of four separate experiments. Effect of OGR1 deficiency was significant (*p < 0.05).
(TIFF)
