# Supplementary figures and images for: Proton-Sensing Ovarian Cancer G Protein-Coupled Receptor 1 on Dendritic Cells Is Required for Airway Responses in a Murine Asthma Model
Source: PLoS One. 2013 Nov 11;8(11):e79985. doi: 10.1371/journal.pone.0079985 (PMC3823589; doi:10.1371/journal.pone.0079985)

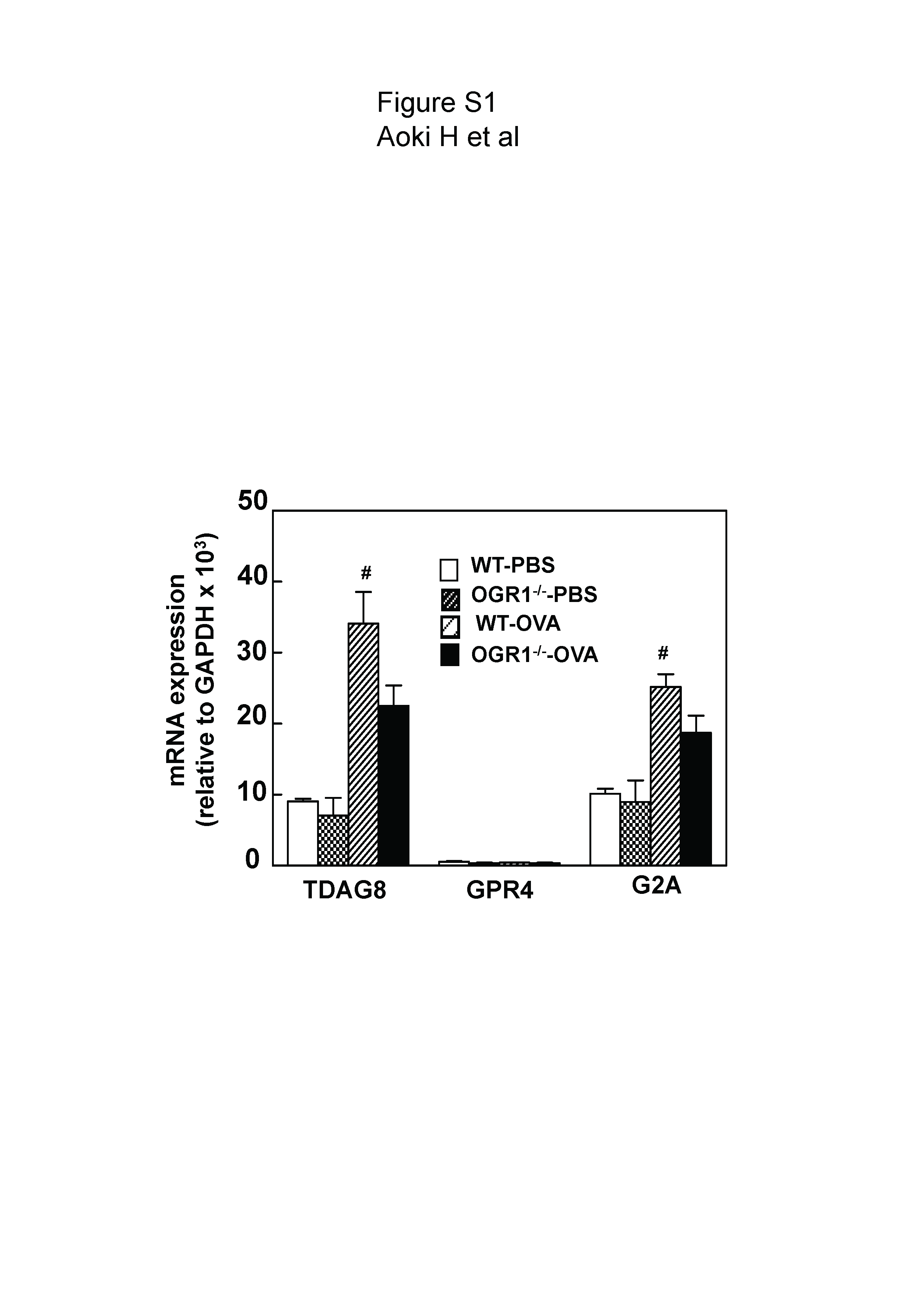

Supplement: Figure S1 — Expression profile of proton-sensing GPCR mRNA in DCs. BMDCs derived from WT or OGR1-deficienct mice were pulsed with PBS or OVA, and expression of TDAG8, GPR4, and G2A mRNAs was evaluated by a quantitative real-time TaqMan PCR. Data are mean ± SEM of three separate experiments. Effect of OVA-priming (WT-PBS vs. WT-OVA) was significant (# p < 0.05). (TIFF) [file pone.0079985.s001.tiff]

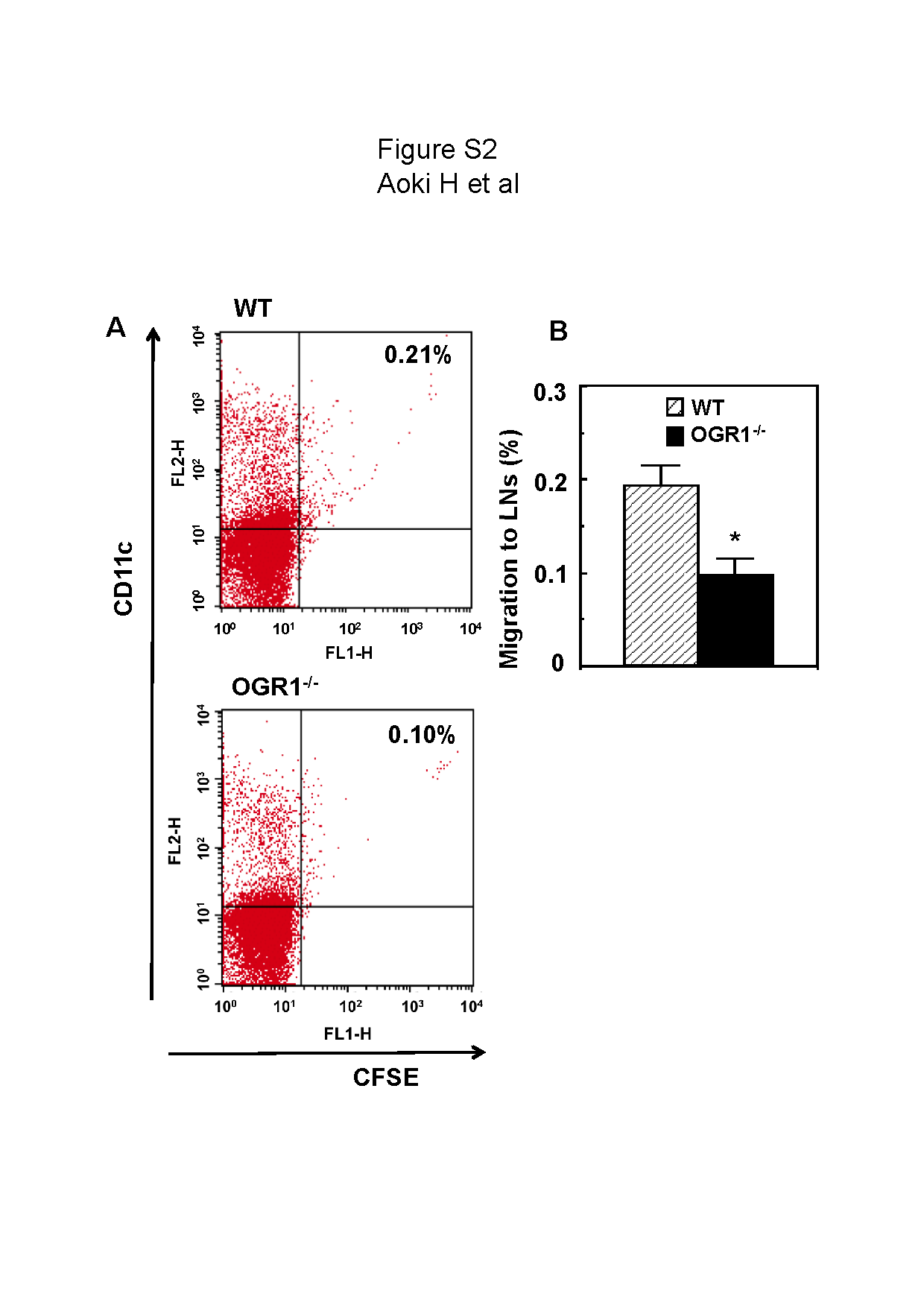

Supplement: Figure S2 — DC migration to popliteal lymph nodes is attenuated by OGR1 deficiency in vivo. CFSE-labeled OVA-pulsed BMDCs from WT and OGR1 -/- were subcutaneously injected to footpads of WT mice. Twenty-four h later, the CFSE+ DCs migrating in draining lymph nodes was analyzed by flow cytometry. (A) Cells recovered from the lymph nodes were assessed for the expression of CD11c and CFSE. Representative flow cytometry plots from 4 separate experiments per group are shown. (B) Percentages of CFSE+DCs (CFSE+CD11c+) per total cells applied were shown. Data are mean ± SEM of four separate experiments. Effect of OGR1 deficiency was significant (*p < 0.05). (TIFF) [file pone.0079985.s002.tiff]
